# Comparing the Performance of Indoor Localization Systems through the EvAAL Framework

**DOI:** 10.3390/s17102327

**Published:** 2017-10-13

**Authors:** Francesco Potortì, Sangjoon Park, Antonio Ramón Jiménez Ruiz, Paolo Barsocchi, Michele Girolami, Antonino Crivello, So Yeon Lee, Jae Hyun Lim, Joaquín Torres-Sospedra, Fernando Seco, Raul Montoliu, Germán Martin Mendoza-Silva, Maria Del Carmen Pérez Rubio, Cristina Losada-Gutiérrez, Felipe Espinosa, Javier Macias-Guarasa

**Affiliations:** 1 ISTI Institute of CNR, Pisa 56124, Italy; Paolo.Barsocchi@isti.cnr.it (P.B.); michele.girolami@isti.cnr.it (M.G.); Antonino.Crivello@isti.cnr.it (A.C.); 2 Electronics and Telecommunications Research Institute (ETRI), Daejeon 34129, Korea; sangjoon@etri.re.kr (S.P.); sylee@etri.re.kr (S.Y.L.); jaehyun.lim@etri.re.kr (J.H.L.); 3 Centre for Automation and Robotics, CSIC-UPM, Arganda del Rey 28500, Spain; antonio.jimenez@csic.es (A.R.J.R.); fernando.seco@csic.es (F.S.); 4 Institute of New Imaging Technologies, Universitat Jaume I, Castellón de la Plana 12071, Spain; jtorres@uji.es (J.T.-S.); montoliu@uji.es (R.M.); gmendoza@uji.es (G.M.M.-S.); 5 University of Alcalá, Department of Electronics, Alcalá de Henares 28871, Spain; mcarmen.perezr@uah.es (M.D.C.P.R.); cristina.losada@uah.es (C.L.-G.); felipe.espinosa@uah.es (F.E.); javier.maciasguarasa@uah.es (J.M.-G.)

**Keywords:** indoor localization, indoor navigation, indoor competition, standard evaluation metrics, benchmarking, performance evaluation, Active and Assisted Living, smartphone sensors, pedestrian dead reckoning

## Abstract

In recent years, indoor localization systems have been the object of significant research activity and of growing interest for their great expected social impact and their impressive business potential. Application areas include tracking and navigation, activity monitoring, personalized advertising, Active and Assisted Living (AAL), traceability, Internet of Things (IoT) networks, and Home-land Security. In spite of the numerous research advances and the great industrial interest, no canned solutions have yet been defined. The diversity and heterogeneity of applications, scenarios, sensor and user requirements, make it difficult to create uniform solutions. From that diverse reality, a main problem is derived that consists in the lack of a consensus both in terms of the metrics and the procedures used to measure the performance of the different indoor localization and navigation proposals. This paper introduces the general lines of the EvAAL benchmarking framework, which is aimed at a fair comparison of indoor positioning systems through a challenging competition under complex, realistic conditions. To evaluate the framework capabilities, we show how it was used in the 2016 Indoor Positioning and Indoor Navigation (IPIN) Competition. The 2016 IPIN competition considered three different scenario dimensions, with a variety of use cases: (1) pedestrian versus robotic navigation, (2) smartphones versus custom hardware usage and (3) real-time positioning versus off-line post-processing. A total of four competition tracks were evaluated under the same EvAAL benchmark framework in order to validate its potential to become a standard for evaluating indoor localization solutions. The experience gained during the competition and feedback from track organizers and competitors showed that the EvAAL framework is flexible enough to successfully fit the very different tracks and appears adequate to compare indoor positioning systems.

## 1. Introduction

In this new digital era, where location-based services are essential in many applications and the number of devices with Internet connectivity has increased dramatically, research on the topic of indoor positioning is flourishing. In contrast to outdoor positioning and navigation, where Global Navigation Satellite System (GNSS) is a mature technology and might be considered a de facto standard, indoor positioning is still in its infancy. The main reason is that GNSS is widespread and is able to provide robust solutions for many applications in a wide range of performance/price ratios, while indoor positioning solutions are few, each specialised for a given scenario and generally expensive.

Many different technologies are being considered for indoor positioning, including Inertial Sensors [1,2,3,4,5], Radio Frequency using RFID, BLE, WiFi or UWB ranging [6,7], Ultrasound [8,9,10], Computer Vision [11], and Light [12,13,14,15]. This diversity in technologies has a significant consequence: there is no generally accepted solution for indoor positioning and navigation. In other words, there is not an indoor equivalent to the GNSS-based portable navigation devices commonly used outdoors. Nevertheless, there are already many companies offering indoor positioning solutions and the forecasts estimate an indoor positioning market of more than USD 20 billion by 2021 [16].

Although the expected commercial impact of indoor location solutions is very high, there is still a lack of standardization in the design, application areas, or evaluation metrics for a fair comparison and assessment of the most promising proposals, both in the literature and at the commercial level. As an example, most of the existing works in the literature show the results of the experiments that were carried out in the authors’ own research facilities, typically department corridors and small research laboratories, which were selected as representative real-world scenarios. Those facilities are not commonly open to other researchers to reproduce their experiments in them. In general, comparing existing working systems is not a trivial task if they have used different technologies, have been evaluated in different scenarios or their performance metrics are not equivalent.

Despite the evaluation heterogeneity found in the literature, some surveys can be found which try to give a consistent overview in this field [17,18,19]. However, most of them show the results as provided by developers, which often do not correspond to a common metric, meaning that an exhaustive comparison cannot be performed.

The research community feels the need for accepted methods of measuring the performance of the proposed indoor positioning systems, devices and technologies [1,18,20,21,22]. In fact, one can observe rising interest in providing open-access datasets, proposing benchmarking tools and, even, organizing competitions in order to facilitate comparative studies.

The EvAAL framework [21] has already been shown to be a consistent way for measuring the performance of personal navigation-oriented positioning systems in indoor environments with known characteristics and, therefore, for rigorously comparing indoor localization systems [21,23,24,25]. These studies back its ambition of becoming a standard in this field.

Although this framework originally aimed at establishing benchmarks and evaluation metrics for comparing Active and Assisted Living solutions, positioning accuracy was an important evaluation criterion. The 2016 Indoor Positioning and Indoor Navigation (IPIN) conference integrated the EvAAL community and framework in the IPIN Competition to compare indoor positioning systems due to their solid experience in organizing competitions. Specifically, three different scenarios were considered in the IPIN 2016 Competition: positioning of people in real-time, positioning of people off-line and robotic positioning. Despite the fact that the robotic positioning might not be related to AAL, it is of interest for the indoor positioning navigation community and it was integrated into the IPIN competition. Therefore, the competition considered different dimensions in the scenario space: pedestrian versus robotic navigation; on-site versus off-site evaluation; usage of smartphones versus customised sensing platforms; infrastructure-based versus infrastructure-free environments. The IPIN community used a well-known evaluation framework in its competition, whereas the EvAAL community had the opportunity to apply their framework in a challenging competition and in other contexts (e.g, the robotic track).

The main objective of this paper is twofold: on the one hand, this paper studies the diversity and heterogeneity of location solutions (different environments, sizes, sensor data and databases, used hardware, etc.) and a review of the different approaches used to evaluate them as proposed by the authors, or by international competitions. On the other hand, this paper aims at demonstrating how the EvAAL benchmarking framework [21] can be used for multiple scenarios and data streams under the large repertory of evaluation tracks at the 2016 IPIN Competition (variety in person/robots, smartphone/custom hardware, multi-storey building, on-line/off-line processing, etc.). We want to demonstrate that a unique evaluation framework, such as EvAAL, can be fitted to many different indoor location use cases.

The remainder of this paper is organized as follows. Section 2 introduces the evaluation heterogeneity found in the literature and the related work aimed at fair benchmarking and comparisons. Section 3 generally describes how the EvAAL criteria were applied to the IPIN 2016 Competition. Section 4 describes the features of the individual competition tracks and shows the results obtained by competitors. Section 5 introduces some lessons learned from the IPIN 2016 competition and the consequent conclusions.

## 2. Diversity Problem Review

This section is devoted to showing the different problems that arise when trying to compare different results from a large diversity of scenarios, different sensor data streams and non-standardized evaluation methods. The next subsections will present the diversity or heterogeneity problem when presenting research location solutions, public databases for testing new algorithms, and the problem related to the diversity of competition conditions and set-ups.

### 2.1. Diversity in Research Systems and Metrics

Some criteria to evaluate Indoor Positioning Systems (IPSs) for personal networks were proposed in [18], for example, privacy, cost, performance and robustness. The authors of [18] observed that the two main performance parameters are the accuracy and the precision, where the former is related to the geometric error and the latter was defined as the success of the position estimations with respect to a predefined accuracy (e.g., the space-based location or the percentage of error in positioning below a threshold). In general, the evaluation metrics depend on the author.

RADAR [20], the first Wi-Fi based indoor positioning system, used the quartile values of the error in positioning (defined as the Euclidean distance between the actual and estimated positions) in order to compare the proposed method to other naïve solutions in a basic analysis. The experimental testbed was located on the second floor of a 3-storey building, with an area of 43.5 m by 22.5 m, more than 50 rooms and three Wi-Fi Access Points (APs). HORUS [26], another well-known IPS developed in 2003 by Youseff et al., provided the median error in positioning and it was tested in two environments: an area of 68 m by 26 m with 172 locations and 21 APs, and an area of 36 m by 12 m with 110 locations and six APs. The two testing approaches are quite different because the density of APs was 0.003 APs/m^2^ in RADAR, whereas HORUS provided two scenarios with densities of 0.012 APs/m^2^ and 0.14 APs/m^2^ respectively, i.e., the scenarios where HORUS was tested had approximately four times the AP density of the evaluation testbed of RADAR. Therefore, comparing the results provided in the original references might not be fair.

In order to provide a fair comparison of RADAR (the deterministic method developed by Microsoft) and HORUS (the probabilistic technique developed by the University of Maryland), they were both implemented and evaluated using the same testbeds [26,27]. According to the data provided in [26,27,28], the density of access points in the testbed used in [26], a university department corridor, was higher than in [27,28]. This change might be due to an improvement of the Wi-Fi network. Despite this minor change in one of the evaluation testbeds, the work done in [26,27] showed that a fair comparison requires the use of the same testbed, or testbeds, rather than reusing the results provided in the literature.

Apart from the diversity of environments, there is also diversity in the hardware elements used for localization and in the metrics to evaluate an IPS. A brief resume of a few works presented in the 2016 Indoor Positioning and Indoor Navigation conference (October 2016, Alcalá de Henares, Spain) is shown in Table 1, where the conference session, base technology, evaluation set-up and evaluation metric are also shown.

According to Table 1, each research work uses specific hardware even when they use the same base technology for positioning. A Huawei Mate smartphone was used in [37], six different devices were used in [38], and a simulation was carried out in [41]. Using multiple devices in the set-up might be more challenging than using a single device or simulated data. Therefore, comparing the results of those works might not be easy at all.

Although the vast majority of papers agree that the error in positioning is defined as the shortest distance between the estimate and current position, consensus in using a particular metric to evaluate the IPS has not been reached yet, as shown in Table 1. This table shows that many metrics have been used to evaluate a IPS, but there is no clear winner metric used in all papers. This conclusion is in line with the study where 195 papers of the first edition of the Indoor Positioning and Indoor Navigation (IPIN 2010) Conference were analysed [21].

For instance, the results of [29,33] are based on the final drift (the geometric error at the end of the track) and visual analysis of the track. The work presented in [46] reports the average error per axis and angle, and the positioning error is not computed. The work presented in [40] shows some plots with measured probability and cumulative distribution instead of providing simple evaluation numbers. Estimated versus real trajectory are the results shown in [34].

Moreover, the scenarios found in the literature are very diverse: from a small 1 m^2^ area or a simulated 3 m by 3 m scenario to a large university hospital or a set of tracks with about 60 accumulated kilometres. Even for the same topic and base technology, the evaluation metrics and the environment depend on the author.

This diversity of evaluation set-ups is not only attached to the IPIN conference; it is a generalized problem in the field of indoor positioning:

- GETA Sandals [55,56] have been evaluated in a few scenarios (straight track, rectangular loop, and climbing stairs) and the results have been provided as the average drift (positioning error at the end of the track) of many users and by plotting the walking distance vs. the positioning error.
- MoteTrack [57,58] evaluation was focused on understanding how it performed under various parameter conditions and the authors carried out different tests. The results were provided as CDFs and plots based on the error distance and a few percentile values.
- Out of Sight [59] is a toolkit for tracking occluded human joint positions based on Kinect cameras. Some in-room test were run for evaluating different contexts (stationary, stepping, walking, presence of obstacle and oclusion). The mean error on the three axes (x, y, z) and the mean positioning error were provided.
- A Kalman filtering-based localization and tracking for the IoT paradigm was proposed in [60], where a simulation in a 1000 m by 1000 m sensor field was performed. The results and comparisons were based on the trajectory plots, and the location and velocity errors in the x and y axes over time (track).
- A smartphone-based tracking system using Hidden Markov Model pattern recognition was developed in [22]. They carried out their experiments in the New Library of Wuhan University using three different device models with over 50 subjects walking over an aggregate distance of over 40 km. The CDF and mean accuracy were used to report the results.

The evaluation heterogeneity present in the literature does not mean that the results shown are not valid; it just makes it more difficult to directly compare the proposed methods with just the information and experiments provided by the authors. The rest of this section will show some initiatives and frameworks for fair evaluation of IPS.

### 2.2. Diversity in the Results Reported in Surveys

Many surveys show comparative studies about indoor positioning technologies (e.g., [17,18,19,61,62,63]). The reported accuracy is often generic and sometimes is reported just as, e.g., ’room level accuracy’, or 50% within around 2.5 m or as a simple range (2 to 5 m). In general, surveys discuss the general accuracy of positioning technologies and the future trends rather than trying to rank the analysed solutions.

Liu et al. [17] introduced a survey about indoor positioning algorithms (angulation, lateration, scene analysis and proximity), performance metrics (accuracy, precision, complexity, robustness, scalability and cost) and technology solutions (GPS, RFID, WLAN, Bluetooth, UWB, cellular).

Gu et al. [18] gave a comprehensive survey of some commercial solutions and research prototypes, which were compared by using many different features: security and privacy, cost, performance, robustness, complexity, user preferences. Trade-offs among the features and the viewpoint of a user in a personal network were also outlined.

Ficco et al. [19] introduced a novel hybrid positioning system. They included an interesting related-work section where 29 different systems, with different base technologies and accuracy ranges, were surveyed.

Xiao et al. [61] offered a comprehensive state-of-the-art survey on wireless indoor localization technologies from the device perspective (device based vs. device-free) in terms of accuracy, cost, scalability and energy efficiency.

He and Chan [62] introduced a survey of Wi-Fi fingerprinting methods, which described advances in two major areas: advanced localization techniques and efficient system deployment. Different methods exploiting spatial and temporal signal patterns were compared according to indoor site availability, additional information for localization estimation, limitations, and reported mean accuracy. Similarly, recent approaches for motion-assisted Wi-Fi localization and typical schemes of collaborative localization were also compared.

Hassan et al. [63] performed a survey on indoor positioning using visible LED light. In a first comparison, they provided general information about other positioning technologies (Wi-Fi, BLE, GSM, among others): accuracy, robustness, complexity, cost and infrastructure reusability. Even for the surveyed LED solutions, they provided an accuracy range in most of the cases.

In general, surveys have one common feature, that is, the IPS are compared using the performance as provided by the authors themselves. Moreover, the metric used to compare accuracy might differ, e.g., the performance of RADAR is 3–5 m in [19], 4 m with 50% probability in [18], and 3–5 m (2.5 with 50% of probability and 5.9 with 90% of probability) in [17]. Results shown in survey works are useful to show the differences between different indoor location technologies, but comparing methods relying on the same technology might not be easy in some cases.

### 2.3. Diversity in Datasets

In the machine learning and pattern recognition community, it is common to test new algorithms over a set of well-known datasets. Researchers tend to upload their collected datasets, and the employed software in some cases, to a database platform or to their own website.

While there is no equivalent platform for indoor positioning yet, you can find numerous public data online.

The Community Resource for Archiving Wireless Data At Dartmouth (CRAWDAD) platform hosts data sets about wireless data including data sets for indoor positioning and mobility. Some illustrative data sets follow:

- A Database related to a Wi-Fi-based positioning system on the second floor of an office building on the campus of the University of Mannheim, released in [64]. The operation area was about 57 m × 32 m but only 221 square meters were covered. The test environment had twelve APs. Seven of them were administered by the university technicians, whereas the others were installed in the nearby buildings and offices. Thirteen additional access points were added for localization purposes.
- Mobility traces at five different sites (NCSU university campus, KAIST university campus, New York City, Disney World -Orlando- and North Carolina state fair) [65].
- Two trace files (one for Wi-Fi and one for Bluetooth) collected by the University of Illinois Movement (UIM) framework using Google Android phones [66].
- One data set to aid the development and evaluation of indoor location in complex indoor environments using round-trip time-of-flight (RToF) and magnetometer measurements [67]. It contains RToF and magnetometer measures taken in the 26 m × 24 m New Wing Yuan supermarket in Sunnyvale, CA, USA. The data was collected during working hours over a period of 15 days.
- The database donated in [68] contains the RSS (Radio Signal Strength) data collected with a mobile robot in two environments: indoor (KTH) and outdoor (Dortmund). The RSS metric was used to collect the RSS data in terms of dBm. The mobile robot location was recorded using odometry (dead reckoning).

The server at the University of California Irvine is a well-known repository of databases for machine learning and pattern recognition problems, the *UCI Machine Learning Repository* [69], where a few datasets for indoor positioning can be found:

- The *UJIIndoorLoc database* is a Wi-Fi fingerprinting database collected at three buildings of the Jaume I university (UJI) campus for indoor navigation purposes. It was collected by means of more than 20 devices and 20 people [70] and was used for the off-site track of the 2015 EvAAL-ETRI Competition [25,71].
- The *UJIIndoorLoc-Mag database* was a magnetic field-based database collected at one laboratory at the Jaume I university (UJI) campus for indoor navigation purposes [72,73].
- The *Indoor User Movement Prediction from RSS Data Set* represents a real-life benchmark in the area of Active and Assisted Living applications. The database introduces a binary classification task, which consists in predicting the pattern of user movements in real-world office environments from time-series generated by a Wireless Sensor Network (WSN) [74].
- The *Geo-Magnetic field and WLAN dataset for indoor localisation from wristband and smartphone data set* contains Wi-Fi and magnetic filed fingerprints, together with inertial sensor data during two campaigns performed in the same environment [75].

The new trend is to publish the databases on the research group website or, even, on dedicated web platforms.

- The Signal processing for the wireless positioning group (Tampere University of Technology, Finland), provides an open repository of source software and measurement data on their website (http://www.cs.tut.fi/tlt/pos/Software.htm). Indoor WLAN measurement data in two four-floor buildings for indoor positioning studies are provided. The data contains the collected RSS values, the Access Points ID (mapped to integer indices) and the coordinates; both the training data and several tracks for the estimation part are provided as indicated in [76,77]. Other datasets with GSM, UMTS and GNSS data are also provided.
- PerfLoc [78] is also running a competition about developing the best possible indoor localization and tracking applications for Android smartphones. The competition participants are required to use a huge database collected by The National Institute of Standard and Technology (NIST) by means of four different Android devices.

On the one hand, using data sets is useful for the research community: the IPS can be evaluated without surveying and mapping; the experiments can be reproduced; and the results can be compared when the same metric is used for evaluation.

On the other hand, a data set has a few disadvantages: it commonly covers one or a few positioning technologies; external researchers (users) do not have control over data collection; some extended information (maps, obstacles, among other useful information from the environment) might not be present; and the data format and the training/validation/test separation depends on the creator/donor.

### 2.4. Diversity in Competitions

In this subsection, we present the problem related to the heterogeneity or diversity of different competition tracks proposed in the last few years for indoor positioning. We mainly review the Microsoft and IPIN competition, describing the diversity of scenarios, data streams and hardware used, and the need for a standardization framework.

#### 2.4.1. The Microsoft Competition

The Microsoft Indoor Localization Competition at the International Conference on Information Processing in Sensor Networks (IPSN) [79] is one of the most popular indoor localization competitions. It held its first and second editions in Berlin and Seattle, in 2014 and 2015. The third edition was held in Vienna in 2016, where the evaluation site was a hall of dimensions 30 by 15 m (450 m^2^).

In the 2016 edition [80], two different tracks were held, called “2D” and “3D”, where position data (XY or XYZ, respectively) had to be generated by competitors at each of 15 different evaluation points. A total of 49 teams showed interest in the competition, of which 35 were registered and 28 were evaluated, 12 in the 2D track and 16 in the 3D one.

In the 2D track, competitors were not allowed to install any devices in the testing hall. Three Wi-Fi APs where available for use. Competitors could use Wi-Fi-based location systems or self-contained solutions using an inertial measurement unit or a magnetometer on the competitor’s body (e.g., a smartphone alone or with some external IMUs).

In the 3D track, competitors were required to install their own hardware, for a maximum of five anchors, in order to estimate the 3D position of the same 15 ground-truth points. Competitors mainly opted for UWB, ultrasonic and laser solutions.

All teams had a whole day prior to the competition for calibration. During the competition, some teams were excluded from the awards since some of them (those based on dead-reckoning such as IMU-based approaches) needed an initial known position. Other teams were also excluded because their system was not real-time, as they needed post-processing.

The main characteristics of the Microsoft competition were kept the same during its first three editions: lack of predefined track formats at the time of subscriptions as formats were precisely defined later, based on competitor’s interest declared during the subscription; the testing environment was about the size of a big hall, with few or no walls, on a single floor; measurements were done in sequence at a series (about a dozen) of fixed points, where the competitors bring their equipment in turn, let the system stabilise, and one organiser reads coordinate estimates on the screen of the competitor’s PC; the metric was the mean of errors, defined as the Euclidean distance between the reference points and the competitor’s estimates.

#### 2.4.2. The IPIN Competition

The International Conference on Indoor Positioning and Indoor Navigation (IPIN) is the oldest conference in this field. IPIN decided to launch its first competition in 2014 and it adopted the EvAAL framework. IPIN formally adopted the EvAAL framework in 2015 and 2016. The main features of the past IPIN Competitions were:

- IPIN competitions take measurements while naturally moving through a predefined trajectory unknown to competitors, instead of evaluating the competitors by standing still at evaluation points;
- IPIN competitions are generally done in big challenging multi-floor environments, possibly on multiple buildings, with significant path lengths and duration, instead of evaluating competitors in small single-floor environments;
- IPIN competitions generally require the competitors to interface with an independent real-time measurement application and test on an independent actor;
- The final score metrics is the third quartile of the positioning error in IPIN, which makes the accuracy results less prone to the influence of outliers and more in line with demanded accuracy for commercial systems.

The entry barrier to IPIN is high, mostly because high stability and robustness are required of the competing systems, which must be able to work reliably for a longer time in tougher conditions. Overall, the EvAAL criteria impose rigorous and realistic test conditions which are appropriate to deal with state-of-the art working systems.

#### 2.4.3. The Scenarios in the IPIN 2016 Competition

The IPIN competition refers to a typical agent indoor navigation scenario, according to which an agent, roaming within an unknown environment, needs to know its current position. The agent moving within the environment can be either a person or a robot. In both cases, we assume that the agent carries an indoor positioning device, e.g., a smart phone or a custom device. For human agents, we refer to a wide indoor environment, such as a mall, a hospital or a big office in which navigation is not a simple task. For robotic agents, we refer to a high precision system. Due to heterogeneity of technologies and target applications, the IPIN 2016 competition includes three different scenarios:

- positioning of people in real time;
- positioning of people off-line;
- robotic positioning in real time.

The first two scenarios aim at assessing the performance of localization systems targeted to humans. The third scenario refers to systems targeted to robotic agents. Competition details are available online at http://evaal.aaloa.org/2016/competition-home.

Scenario 1: Positioning of people in real time 

**Figure 1:**
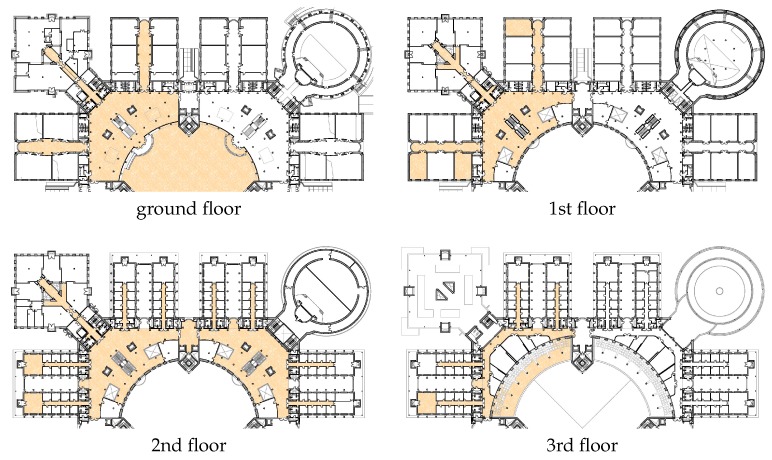
Floormaps of the IPIN 2016 competition: Positioning of people in real time.

The goal of this scenario is to measure the performance of working systems with people moving in realistic conditions. The setting was the Polytechnic School of the University of Alcalá (EPS-UAH), which is divided into four sectors (red, blue, green and yellow) of which two contiguous ones (the red and blue sectors) were used in the competitions for positioning people in real time. Figure 1 shows the floor plans of this scenario, where the usable areas are marked with an orange pattern.

In order to cover different positioning technologies, two tracks were proposed to competitors.

- Track 1: Smartphone-based (on-site);
- Track 2: Pedestrian dead reckoning positioning (on-site).

The main differences between these two tracks are the positioning devices allowed.

Scenario 2: Positioning of people off-line 

Like the previous one, the purpose of this scenario is to measure the performance of working systems with people moving in realistic conditions. The difference is that the competition is performed off-line. The calibration and evaluation data to set up and check the indoor positioning systems are provided in advance to competitors by organizers. Therefore, all competitors have the same data and information to create and evaluate their systems.

**Figure 2:**
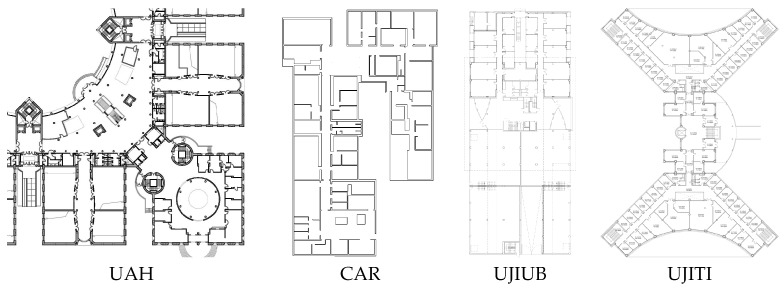
Floor maps of the IPIN 2016 competition: Positioning of people off-line.

Since evaluation time is not a tough restriction, the setting was composed by many different tracks which were pre-recorded in four heterogeneous buildings: the green sector of the Polytechnic School of the University of Alcalá (EPS-UAH), the CAR building (CSIC Arganda, Madrid), and UB & TI buildings (Universitat Jaume I, Castellon). The setting at EPS-UAH building was different from Tracks 1 and 2, in order to avoid interfering with them. Figure 2 shows the base floor plans (only ground floor for simplification) of the four buildings used for this scenario. The databases of the off-site track are available online at http://indoorloc.uji.es/ipin2016track3/.

In this scenario, only one track was available to competitors:

- Track 3: Smartphone-based (off-site)

Scenario 3: Robotic positioning 

**Figure 3:**
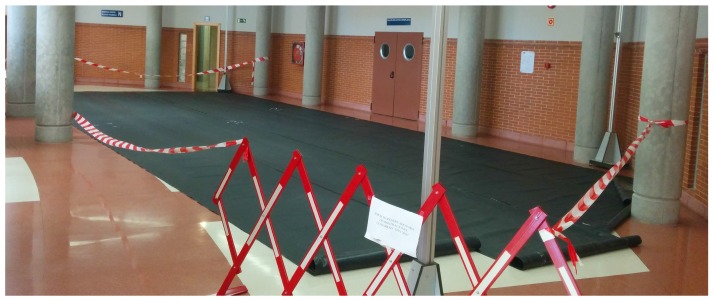
Robotic positioning area at IPIN 2016 Track4 test. The ground-truth track is hidden by a black cover.

The purpose of the last scenario is to continuously log the trajectory followed by an industrial robot. Robots are generally programmed to accomplish specific tasks, e.g., delivering postal mails among offices or delivering medical supplies to laboratories in a hospital. In the competition scenario, the industrial robot followed a closed path, including circular and straight sections alternately arranged. The path was unknown to participants in advance, as shown in Figure 3. Competitors provided both their own custom hardware, that had to be deployed and calibrated in a constrained time, and their algorithm approaches for tracking the robot trajectory.

In this scenario, only one track was available to competitors:

- Track 4: Indoor mobile robot positioning (on-site)

#### 2.4.4. Other Competitions

Robotics competitions have a large tradition, such as the Robot World Cup (Robocup) [81], the Urban Search and Rescue (USAR) [82,83] or the AUVSI SUAS Competition for Unmanned Aerial Systems (UAS) [84]. These competitions play an important role in the development of artificial intelligence (AI) and robotics, but none of them is focused on indoor robot positioning.

## 3. EvAAL Framework Applied to IPIN Competition

In this section, we first review some of the most prominent benchmarking metrics proposed to date (EVARILOS and EvAAL), and then we show how the EvAAL framework has been integrated into the IPIN competition to evaluate diverse positioning systems under multiple scenarios (multi-floor, multi-building, multi-sensor, multi user persons/robots, etc.) in order to create a de facto standard for evaluation.

### 3.1. Benchmarking Metrics

#### 3.1.1. The EVARILOS Benchmarking Framework

The EVARILOS benchmarking platform [85], with a competition held in 2014, had the objective of automating the evaluation process, although the final error metrics were calculated with a manual process from the ground truth. The EVARILOS benchmarking proposal defines a taxonomy of metrics (deployment, functionality and performance related) integrated in a weighted sum to finally generate the system scores. Although it targets radio frequency-based systems, some of their ideas could be generalized to broader scenarios.

#### 3.1.2. The EvAAL Benchmarking Framework

The first public international localization competitions (2011–2013, Valencia and Madrid) were part of the EvAAL initiative [21,23]. The EvAAL framework is characterised by several core criteria in order to evaluate the teams:

1. *Natural movement of an actor*: the agent testing a localization system is an actor walking with a regular pace along the path. The actor can rest in a few points and walk again until the end of the path.
2. *Realistic environment*: the path the actor walks is in a realistic setting: the first EvAAL competitions were done in living labs.
3. *Realistic measurement resolution*: the minimum time and space error considered are relative to people’s movement. The space resolution for a person is defined by the diameter of the body projection on the ground, which we set to 50 cm. The time resolution is defined by the time a person takes to walk a distance equal to the space resolution. In an indoor environment, considering a maximum speed of 1 m/s, the time resolution is 0.5 s. These numbers are used to define the accuracy of measurements. When the actor walks, measurements are taken when he/she steps over a set of predefined points. The actor puts his/her feet on marks made on the floor when a bell chimes, once per second. As long as he/she does not make time and space errors greater than the measurement resolutions, which is easy for a trained agent, the test is considered adequate.
4. *Third quartile of point Euclidean error*: the accuracy score is based on the third quartile of the error, which is defined as the 2-D Euclidean distance between the measurement points and the estimated points. More discussion on this is in the next section. Using the third quartile of point errors, the system under measure provides an error below the declared one in three cases out of four, which is in line with the perceived usefulness of the experimental IPS. Reference [86] argues that using linear metrics such as the mean may lead to strange and unwanted behaviour if they are not properly checked because of, for instance, the presence of outliers. A clear example of this behaviour can be seen in [25], where a competitor of the off-site track provided severe errors in very few cases and its average error and root mean squared error were negatively highly affected, however the competition metric, the third quartile, showed that the solution proposed was not as bad as the averaged error had shown. More detailed discussion is found in [21].

The core criteria are the distinguishing features of the EvAAL framework. The first EvAAL competitions additionally adopted the following extended criteria:

1. *Secret path*: the final path is disclosed immediately before the test starts, and only to the competitor whose system is under test. This prevents competitors from designing systems exploiting specific features of the path.
2. *Independent actor*: the actor is an agent not trained to use the localization system.
3. *Independent logging system*: the competitor system estimates the position at a rate of twice per second, and sends the estimates on a radio network provided by the EvAAL committee. This prevents any malicious actions from the competitors. The source code of the logging system is publicly available.
4. *Identical path and timing*: the actor walks along the same identical path with the same identical timing for all competitors, within time and space errors within the above defined resolutions.

### 3.2. Applying EvAAL Criteria to IPIN 2016 Competition

During IPIN 2016, four competition tracks were run in parallel. The competitors of each track were evaluated according to the third quartile of the positioning error. This error is measured based on xy coordinates (longitude and latitude). Also, a *penalty*P=15 m is added for each floor error. For example, if the xy error is 4 m and the estimated floor is 2 while it should be 0, the computed error for that estimate will be 4+2P=34 m. This is in line with real users’ point of view, because some movements in the environment are restricted by physics and the architectural elements [87].

As far as the *core* criteria of the EvAAL framework are concerned, the IPIN competition 2016 follows them as close as possible for a given scenario.

- The two first *core* EvAAL criteria are followed closely: in Tracks 1–3 the actor moves naturally in a realistic and complex environment spanning several floors of one (for Tracks 1 and 2) or few (Track 3) big buildings; in Track 4, the robot moves at the best of its capabilities in a complex single-floor track.
- The same holds for the third core criterion: the space-time error resolution for Tracks 1–3, where the agent is a person, are 0.5 m and 0.5 s, while space-time resolution for Track 4, where the agent is a robot, are ≈1 mm and 0.1 s. In Track 4, only the adherence to the trajectory is considered given the overwhelming importance of space accuracy with respect to time accuracy as far as robots are concerned.
- The last core criterion of the EvAAL framework is followed as well, as the third quartile of the point error is used as the final score. The reason behind using a point error as opposed to comparing trajectories using, for example, the Fréchet distance [38,88] is that the latter is less adequate to navigation purposes, for which the real-time identification of the position is more important than the path followed.

As far as the *extended* criteria of the EvAAL framework are concerned, the IPIN competition also similarly follows them.

- In tracks 1 and 2, the path is kept secret only until one hour before the competition begins, because it would be impractical to keep it hidden from the competitors after the first one in a public environment. However, competitors could not add this knowledge to their systems. In Track 3, the competitors work with blind datasets (logfiles) in the evaluation so the path can be kept secret. In Track 4, a black cover is used to avoid any visual reference of the path and other visual markers.
- The agent is independent for all tracks apart from Track 2, where the technical difficulties of the track suggested that the actor was allowed to be one of the members of the competing team.
- The logging system is only independent in Track 1. An exception was added in Track 2 for the logging system, which was done by the competitors themselves rather than by an independent application. In Track 3, competitors submitted the results via email before a deadline. In Track 4, the competitors had to submit the results via email within a 2-min window after finishing the evaluation track.
- The path and timing was identical for all competitors in Track 3. The path and timing was also identical for all competitors in Track 4. The paths are slightly different in tracks 1 and 2, which involved positioning people in real-time, because the path was so long that it would have been impossible to force the actors to follow exactly the same path with the same timing many times.

## 4. The IPIN 2016 Competition Tracks

In order to see the potential power of the EvAAL framework for evaluating different and diverse locating scenarios and systems, we now give some detail about the three different competition scenarios used in the past 2016 IPIN competition.

### 4.1. Tracks 1 and 2: Positioning of People in Real Time

This subsection details the rules for Tracks 1 and 2 of the IPIN 2016 Competition since they have many commonalities. The competing systems had to be engineered or prototyped so that an external actor could use them without impairing his movements.

In Track 1, actors were not trained to use the competing system. Generally, actors are people from the conference audience or people from the organizing committee willing to support the competition. In Track 1, competitors had to use standard smartphones, with the possibility of using any sensor available on the device: GPS, accelerometer, gyroscope, compass, Wi-Fi radio and barometer. Competitors were allowed to only exploit the existing Wi-Fi access points available on the competition area. Teams in Track 1 were allowed to perform two runs each during the competition day, and consider only the best result. During each run, the actor tested one competing application by using a measurement application developed by the organizers, called StepLogger.

Competitors in Track 2 were allowed to use any IMU sensor module, whether off-the-shelf or self-developed, without any limit concerning the number of devices and the mounting position on the body. Laptops, tablets and smartphones could be used to process data gathered from the Pedestrian Dead Reckoning (PDR) system in real time. Competitors were requested to provide the organizing committee with the sensor raw data, the estimated positions of the system and the key point indexes according to a specific log format. Competitors were not requested to use an external measurement application and could generate the log themselves.

The detailed competition rules are published on the IPIN web page [89,90].

#### 4.1.1. Surveying the Area

Competitors of both tracks received detailed geo-referenced maps of the competing area and were able to survey the area during the days before the competition day. They used the survey time to check how their system performed in the area and were able to perform algorithm tune-up.

During the survey, competitors in Track 1 were mostly interested in taking Wi-Fi measurements, both fingerprints in various locations (a long and tedious task) and checks on the position of access points in the area. Most access points were, in fact, indicated on the maps, but some were not, and up-to-date ids (MAC, SSID) were not provided to competitors. After the competition, many competitors in Track 1 informally said their system would have performed much better if they had spent more time in taking Wi-Fi measurements during the survey, because the area was so big and the hours they dedicated to the task were not enough. Most notably, the winning Track 1 team needed very little survey time. To the amazement of bystanders, including some other competitors, after slightly more than half an hour spent in a specific small area of the building, they claimed that they had collected all the data required by their system.

#### 4.1.2. Evaluation Path

A reference path is necessary to measure the accuracy of the competing systems, and was used as the ground truth. In practice, some dozen markers (keypoints) were stuck on the floor and their coordinates were measured in advance. The actor followed the markers sequentially stepping over them. For Track 1, the synchronization between the real position of the actor and the estimated position was guaranteed by the StepLogger app, running on the same smartphone where the competing app was running. When the actor steps over a mark on the floor, he pushes a button on the smartphone’s screen, and the app records the time. Since the markers are walked in a predefined order, the time stamps are easily associated with the corresponding mark and logged to persistent memory. The log thus produced is then compared with the log of continuously estimated positions provided by the competitor.

In Track 2, the same time-stamp log can be provided by the competitor if he/she uses the StepLogger app. Since most of PDR systems are implemented on laptops, it is not easy to have a communication interface with the StepLogger app, which runs on Android. Therefore, competitors generated logs including time-stamp, sensor raw data, estimated position and marker indexes indicating whether the position value is calculated at the reference point or not.

In both Tracks 1 and 2, the organisers provided an application for producing the results from the time-stamp log and the log of competitor estimates. The applications read the space-time logs and computed the errors as xy distance plus floor penalty. The third quartile of error is then computed and presented as the final score. A dedicated application, available on the EvAAL website, produces a graphical representation of the map including the ground truth and the estimated path.

The path was defined with the goal of realistically reproducing the way people move within wide indoor environments. In fact, path complexity is a distinguishing feature of the IPIN competition. For this purpose, the following rules were considered:

- stairs (for both Tracks 1 and 2) and a lift (Track 1 only) are used to move between floors;
- the path traverses four floors and includes the patio, for a total of 56 key points marked on the floor, 6000 m^2^ indoor and 1000 m^2^ outdoor;
- actors stay still for few seconds in six locations and for about 1 min in three locations; this cadence is intended to reproduce the natural behaviour of humans while moving in an indoor environment;
- actors move at a natural pace, typically at a speed of around 1 m/s;
- total length and duration are 600 m, 15’ ± 2’, which allows to stress the competing apps in realistic conditions.

As mentioned before, the setting was the Polytechnic School of the University of Alcalá (EPS-UAH). EPS-UAH is hosted in a square building composed of four floors connected with stairs and lifts. Floors all have a similar layout, with a side of about 140 m. The central big and open round hall gives access to four wings where medium sized rooms are located. The structure is mostly made of concrete with many pillars in the central hall. Glass walls separate the patio from the ground floor indoor areas. Wi-Fi is available inside and immediately outside the building.

**Figure 4:**
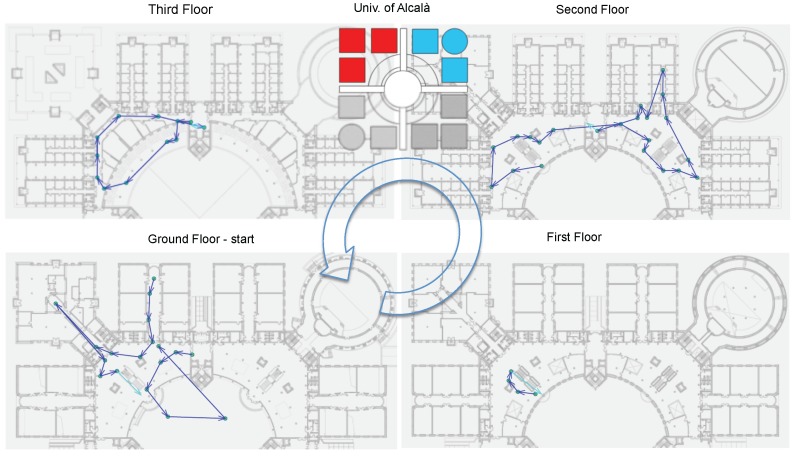
The IPIN competition path for Track 1 and Track 2.

The path is shown in Figure 4. It is composed of 56 key points, five of which were placed in the patio. The path starts from ground level, up to floors 1, 2 and 3 by means of stairs, then goes to a terrace about 50 m long and proceeds to the ground level, goes to the patio and indoors again. When going to the lower floors, the lift is used for Track 1, the stairs for Track 2.

Key points were labelled with a tag in the form [building ID, floor, marker ID] (Key point labels were written on the button displayed by the StepLogger app, to reduce errors on the actor’s part which would require him to restart the path from the beginning). They were placed on the floor following these criteria:

- key points were placed in easily accessible places where people usually step over;
- distance between key points ranged from about 3 to 35 m, with a median of 8 m;
- each key point was visible from the previous one, to ease the movement of the actor and reduce random paths between two consecutive key points.

#### 4.1.3. Track 1 Results

In the first track, six teams were admitted [91,92,93,94,95,96]: NavIndoor, Samsung, WiMag, WMLoc, XMUH and XPosition. Each competitor had two chances to run the path, with only its best performance considered.

**Figure 5:**
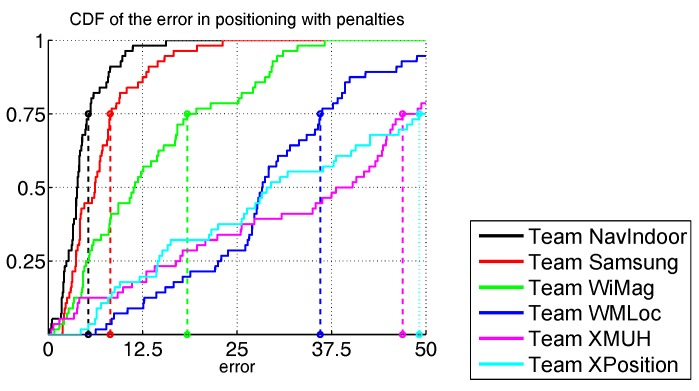
Cumulative distributions of localization error in metres for Track 1.

NavIndoor and Samsung teams obtained the best results, respectively with a third quartile score of 5.4 m and 8.2 m, while the remaining teams had scores higher than 15 m, as shown in Figure 5. Note that the localization errors are in the order of a few metres, which is acceptable for navigating an indoor environment and consistent with the EvAAL criteria. Again, we stress that we tested real working systems in a realistic environment with a realistic usage pattern and rigorous criteria: no simulations, no small or controlled environments, and no simplifying assumptions.

#### 4.1.4. Track 2 Results

In Track 2, a total of eight teams were admitted but finally six of them competed [97,98,99,100,101,102,103,104]: NESL, SYSNAV, PARMA, DEUSTO, XIAMEN and TIANJN. Two trials were given for each team, and the best result was selected for comparison.

**Figure 6:**
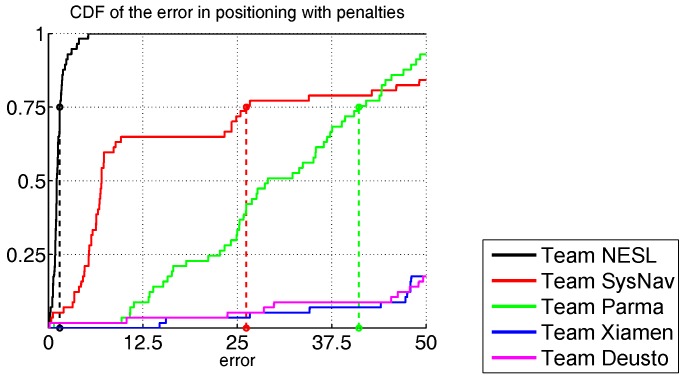
Cumulative distributions of localization error in metres for Track 2.

The results of Track 2 show a high deviation of positioning errors among the six competing teams. NESL produced the best result with a third quartile score of 1.5 m. The second ranked team, SYSNAV, showed very good performance during the initial phase, but the final score was 26.2 m. Such negative performance is due to the sunlight, an unexpected source of error, encountered when the competitor walked along a terrace on the fourth floor. All the other teams produced higher scores than 40 m, as shown in Figure 6, the main cause for this being inaccurate initial heading evaluation. Considering PDR competitors were not allowed to exploit any information gathered from the competition area, such as the Wi-Fi networks, handling of initial heading was a key factor for obtaining reasonable performance.

### 4.2. Track 3: Smartphone-Based (Off-Line)

In contrast with the two previous tracks, this track was entirely run off-line.

The competitors had to use databases provided by organizers to train and calibrate their systems. Those databases contained data from any sensor available on the smartphone used to log the track performed by an external actor (competition organizers and students). The competitors were not allowed to record any additional measurements on-site. Similarly, data for evaluation was also provided by organizers.

The detailed competition rules are published on the IPIN and competition web pages [105,106].

#### 4.2.1. Surveying the Area

The main objective is to evaluate all competitors under exactly the same conditions. Therefore, they were not allowed to survey the scenarios by themselves to collect additional measurements.

Participants received a set of calibration log files with data from smartphone sensors which were recorded on different paths in a total of four different buildings. Different scenarios (four buildings), smartphones (four device models) and actors (eight people) were involved at this stage in order to maximize the heterogeneity of data. A total of 10 routes, considering the four buildings, were selected according to the same realistic-based goals established for the first and second track (see Section 4.1.2), which included 1025 calibration points (key points).

Competitors were asked to design and implement their localization systems exploiting a total of 17 calibration log files. Logs were publicly available since the very beginning of the call to guarantee that all competitors had the same time to set up their systems.

##### Data Format

Competitors used the log files generated by the *GetSensorData* application (http://lopsi.weebly.com/downloads.html) in which a user walks along a predefined path. Logs are text files where each row contains data received from a different sensor available on the the phone at a given time. Each row begins with the sensor identifier, as four capital letters, that determines the kind of sensor data (Wi-Fi, Accelerometer, Magnetometer, etc.). Then, the timestamps and sensor readings follow, separated by a semicolon. The reference data to train/generate the IPSs was geo-referenced. So, the position of well-known landmarks trough the path were provided as special entries in the log file. For the evaluation log files, explicit location of landmarks was not provided. A simple Matlab-based application was provided to competitors in order to parse and re-arrange the log files (http://indoorloc.uji.es/ipin2016track3/).

#### 4.2.2. The Evaluation Path

Nine evaluation trajectories, or paths, were defined with the goal of realistically reproducing the way the people move within wide indoor environments, as was done for the evaluation in Tracks 1 and 2. As mentioned before, the high path complexity is a distinguishing feature of the IPIN competition. For this purpose, the following rules were considered:

- the stairs and the lifts could be used to move between floors;
- the paths traverse four floors in the UAH building, one floor in the CAR building, six floors in the UJIUB building and four floors in the UJITI building;
- paths in the CAR building also include an external patio;
- the nine paths cover a total of 578 key points;
- total duration is 2 h and 24 min, which allows to stress the competing applications in realistic conditions;
- actors may stay still for few seconds in a few locations; this rhythm is intended to reproduce the natural behaviour of humans while moving in an indoor environment;
- actors move at a natural pace, typically at a speed of around 1 m/s;
- phoning and lateral movements were allowed occasionally to reproduce a real situation;
- competitors have all the same data for calibrating and competing.

As already mentioned, the setting was composed of four different buildings in different cities. The UAH building is in a university environment with corridors, classrooms, laboratories, among other typical facilities. The UJITI building has similar facilities but the Wi-Fi network infrastructures are different, since UAH and UJI have different strategies to deploy Wi-Fi. Unlike the two previous buildings, CSIC is a research institution and the UJIUB building has many offices hosting technological companies. In general, each of the selected buildings has unique features (occupancy, construction materials, architectural elements, Wi-Fi network policies, among many others).

Competitors tested their localisation algorithms with nine evaluation log files and they sent us the estimated locations in a predefined format. The evaluation log files were not geo-referenced so competitors had to exploit the information collected by all sensors in order to provide an accurate position estimation. It is important to mention that once the evaluation log files were released, all competing teams had strict timing to provide their results.

Since the competitors had just one chance to submit results without feedback to readjust their systems on-the-fly, up to three different results from each participant were accepted in this track, but only the best result was considered for the final ranking. In Tracks 1 and 2, the competitors had two chances, but they had feedback about their accuracy after the first run. The evaluation was performed off-line before the conference.

#### 4.2.3. Results

In Track 3, a total of six competing teams were admitted but only five of them formally registered [91,107,108,109,110]: HFTS, UMinho, m BlockDox, NavIndoor (FHWS in Track 3 to avoid ambiguities with respect to Track 1) and Marauder.

**Figure 7:**
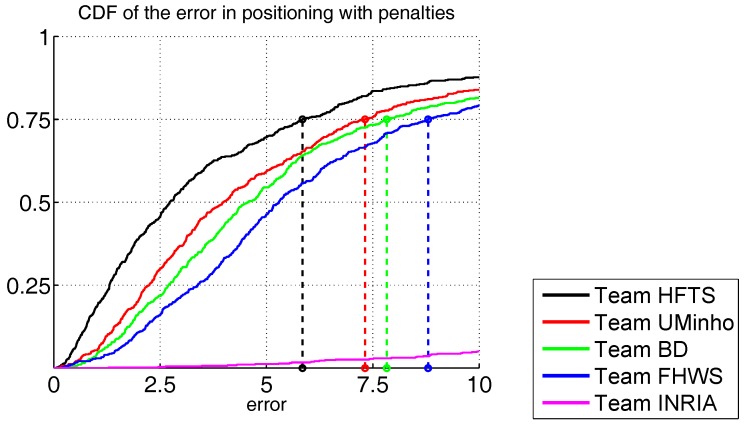
Cumulative distributions of localization error in metres for Track 3.

The HFTS and UMinho teams obtained the best results, with 5.9 m and 7.3 m third quartile. BlockDox and FHWS provided slightly higher scores, at 7.8 m and 8.8 m respectively. The remaining team provided a score higher than 40 m. Results of Track 3 are shown in Figure 7.

It is worth noticing that the localisation errors we experienced are also in the order of meters: this is consistent with Track 1 results.

### 4.3. Track 4: Robotic Positioning

Track 4 is significantly different from other tracks due to three main facts:

1. The tracked element is an industrial robot;
2. The task not only needs discrete and usually well separated key positions to be estimated, but a detailed tracking of the actual precise and unknown robot trajectory;
3. Competitors could put sensors on board as well as locate them on given poles around the navigation area.

Detailed competition rules are published on the IPIN web page [111].

#### 4.3.1. Surveying the Area

Participants decided both the nature and the number of sensors, and had up to 45 min to assemble, mount and calibrate their systems. Three reference points in the scenario were given to allow sensor calibration; one of them was the start/stop point for the mobile unit. Competitors had to report the consecutive points of the robot trajectory at least every 100 ms (minimum frame rate). The required information was the x,y coordinates (in millimetres) with respect to the starting point, and the corresponding time stamp.

##### Robot

**Figure 8:**
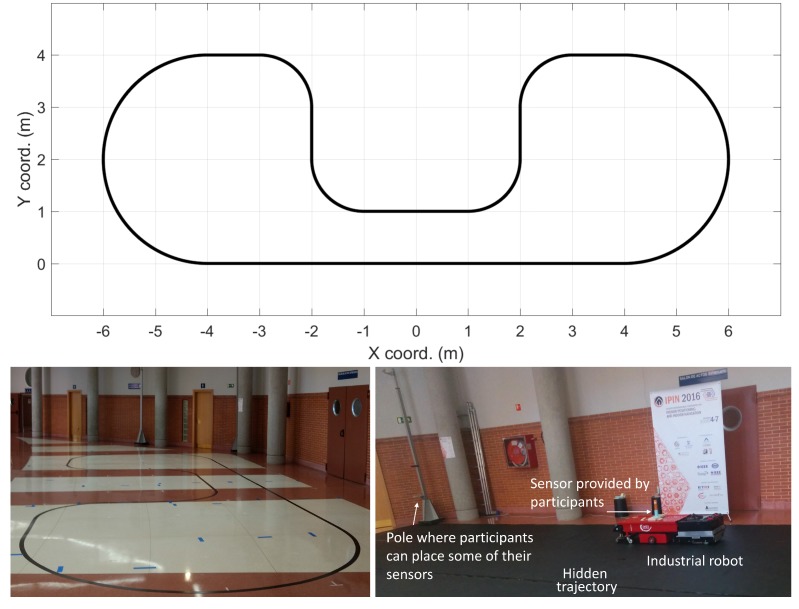
Evaluation path and environment for Track 4.

The chosen mobile unit was a Standard Easybot from the ASTI international robotic company [112] (see Figure 8 bottom-right). The Easybot is an automatically guided vehicle implementing a magnetic guidance and its nominal velocity is 0.6 m/s, tracking the designated magnetic tape thanks to the feedback provided by a magnetic detector and the corresponding closed-loop control.

#### 4.3.2. Evaluation Path

The magnetic path was a closed route including circular and straight sections alternately arranged (see Figure 8 top). The magnetic tape was placed on a trajectory that was previously defined according to a mathematical description; this way, we assured a known ground-truth, identical for every experiment (see Figure 8 bottom-left). The path length was 32.84 m; the trip time was around 3 min, including programmed linear velocities changes, which were equal for all participants. A black cover avoided any visual reference of the magnetic path (see Figure 8 bottom-right). A mark placed on the robot defined the reference point to be tracked by competitors.

#### 4.3.3. Results

Track 4 received six requests for participation from teams using different technologies (UWB, visible light, acoustics and laser), but only five of them formally registered [113,114,115,116,117]. Two of them were local teams from the hosting university (University of Alcalá) and were considered as off-track competitors, three of the remaining groups formally registered, but only two teams competed and were evaluated: the ATLAS [115] team and the TopoRTLS company [114].

**Figure 9:**
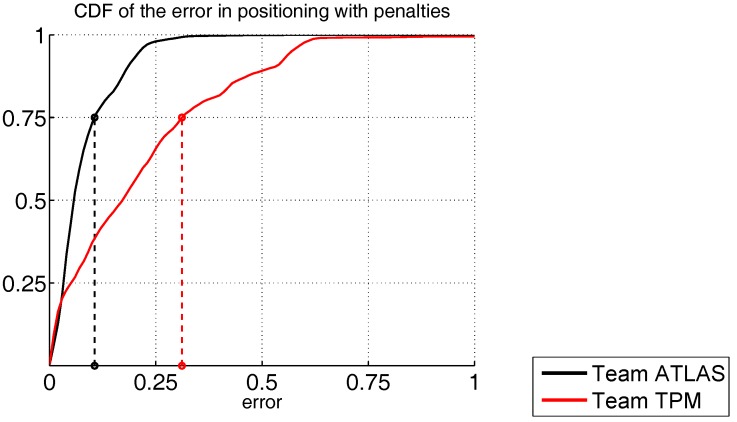
Cumulative distributions of localization error in metres for Track 4.

Figure 9 depicts the CDF of both participants, where the errors at the third quartile were 106 mm and 311 mm for the ATLAS and TPM teams, respectively.

### 4.4. Lessons Learned from IPIN Competition Tracks

The 2016 IPIN competition has been a successful and challenging experience both for the organizers and the competing teams. We can derive some lessons we learned during the preparation of the tracks and also during the competition days.

In smartphone-based Track 1, we observed that tests done on a realistic and long path stress the competing applications, which require a good degree of maturity to run regularly for long time periods. The independent actor may behave differently to how the competitors had anticipated. We think that these are key features for assessing the performance in realistic conditions.

As far as performance is concerned, we noted that accuracy, speed and scope of site surveying, especially for Wi-Fi measurements, was the key to success for all competitors.

In PDR Track 2, we observed that a correct initial heading is important for obtaining good performance: for three teams, a bad initial heading produced a path rotated by some degrees with respect to the ground truth, resulting in bad performance even in the presence of a correctly-shaped path. Therefore, it could be considered to provide competitors not only with the starting point coordinates, but additionally with initial heading information for Track 2. As a second note, quality of sensors turned out to be an important factor. Three teams used commercially available sensors; the others used self-developed sensor modules: the lack of engineering in the latter was sometimes crucial. In one case, a sensor module misbehaved, so the team missed the first trial; in another case, the Bluetooth communication between the sensor module and the user mobile device was unreliable, resulting in incomplete log files.

In the off-line Track 3, we observed that the best results are similar to Track 1, which is a compelling argument for the usefulness of off-line tests. On the other side, the NavIndoor team from FHWS participated in both tracks with the same system, and it reported lower accuracy off-line than in real time. It can be argued that the presence of both real-time and off-line tracks with similar characteristics has enriched the IPIN competition. Furthermore, the data sets are open-access and they can be reused by teams that did not participate in the competition. Therefore, the research community can make new comparisons of state-of-art systems.

In robotics Track 4, the resulting localization accuracy is better than in the other tracks due to the fact that the navigation area is much smaller, the ground truth path has a much higher measurement accuracy and the teams had the possibility of installing their specific equipment on-board and off-board the robot. A consistent and sufficiently precise ground-truth can be achieved by using a mathematically modelled trajectory and considering time sequence information. Similar to the smartphone-based Track 1, time necessary for equipment placement and calibration, which depends on system complexity, has been demonstrated to be an important performance-limiting factor. On the other hand, the competition set-up has to consider enough space for an industrial robot performing a diverse and complex trajectory and competition rules must provide enough description about physical elements available for participants to deploy their systems, together with size, weight and safety constraints.

## 5. Conclusions

We have shown how diverse current indoor location solutions are and how the different environments, different test areas, different sensor technology, etc., have a significant impact on how location results are processed and evaluated, which makes it difficult to directly compare performance.

We gave a review of the ways to approach this problem in the literature, either by using the performance measure indicated by the authors themselves, or by means of a competition. One additional approach to compare algorithms in controlled conditions has been to set up measurement databases; however, differences in formats, recording procedure and range of sensor used again makes this approach not straightforward.

As a possible solution, we propose the use of the EvAAL benchmarking framework, which we claim has the potential to become a standard way to compare systems in different application areas. It has been used since 2011 and lately in the 2016 IPIN competition, where four different tracks were focused on different use cases with significant variation along several dimensions: person vs. robot, smartphone vs. custom hardware, single vs. multi-storey building, single vs. multi-building environments, on-line vs. off-line processing. Our claim is that applying the EvAAL rigorous criteria makes it possible to directly compare the performance of heterogeneous systems in a more significant way than with other existing methods. We back this claim by presenting the details of the different tracks in the IPIN competition and showing how, in their diversity, they are still comparable.

Experience gained during the IPIN 2016 competition and personal feedback from track organizers and competitors encourages the idea that the EvAAL framework is flexible and robust enough to successfully fit very different use cases when comparing indoor positioning systems.

Finally, one of the main challenges of evaluating indoor positioning systems is considering the diversity of contexts and scenarios during the evaluation. In the 2016 IPIN competition, as it has been already mentioned, a few different contexts and scenarios were considered through the four tracks with the assumption of the normal actor’s movement. The spirit of EvAAL is to be open and integrate new tracks whenever it is possible. For instance, one difference with respect to the previous 2015 EvAAL-ETRI competition was the inclusion of robot-based Track 4. Databases to cover many multi-tier diverse buildings were introduced in the 2015 edition. As future work, the EvAAL community is open to discussing the inclusion of new tracks where the actor’s movement might be erratic (e.g., building evacuation in the case of a fire alarm), non-constant (e.g., sport tracking) or presenting non-walking patterns (e.g., people with mobility impairments) which are realistic movements in some contexts.

## Figures and Tables

**Table 1:** Evaluation set-up of papers presented during the 2016 Indoor Positioning and Indoor Navigation conference. PDF is Probability Density Function, CDF is Cumulative Density Function.

Ref.	Session	Base Tech	Evaluation Scenario	Evaluation Metrics
[29]	Hybrid IMU	ifoot-mounted IMU iBeacons Smartphone	1 walking track of 5.4 km	Final error Trajectory
[30]	Hybrid IMU	Smartphone sensor fusion	4-storey building (77 × 55 m) 4 walking tracks	Average Error
[31]	Hybrid IMU	Xsens MTw inertial sensor	multi-storey office building	Average Error Root Mean Square Error
[32]	Hybrid IMU	Data acquisition platform with IMU, UWB and BT	2 rooms and 2 corridors 1 walk	Position error Heading error Trajectory
[33]	Hybrid IMU	visual-magneto-inertial system	multiple experiments: 1 m^2^ area; staircase; and motion capture room	final drift Trajectory
[34]	Hybrid IMU	Google Nexus 5 Sensor fusion	1 walking track	Trajectory
[35]	Hybrid IMU	low-end smartphone low-end tablet	different tests	$F_{1}$ Final Disc. Ratio Average Error Hints
[36]	RSS	no device info	2 rooms	Average Error Root Mean Square Error Max. Error CDF
[37]	RSS	Huawei Mate	Garage	Average Error
[38]	RSS	6 different android devices	Large university hospital >160.000 m^2^ (3 floors)	Average Error Error variants échet distance
[39]	RSS	Competition Database Wi-Fi fingerprinting	3 multi-storey buildings	Average Error Median Error 95th percentile building & floor rate
[40]	RSS	Device not defined	China National Grand Theatre 210 × 140 m	PDF CDF
[41]	RSS	Simulation (RSS)	8 × 8 m	Complex Scatter plot
[42]	RSS	7 smartphone models (50 subjects)	Set of routes ≈50 km + ≈7 km	Average error Histogram
[43]	Magnetic	MIMU Platform [44]	2 walks: Office and mall	Average error Trajectory and ROC curve
[45]	Magnetic	Magnetic and camera: Project Tango and Google Nexus 5X	Noreen and Kenneth Murray Library 2 different floors with strong and weak disturbances	Average error Visual results Matching rate
[46]	Ultrasounds	Senscomp 7000r and proposed HW platform	Not described	Average error by axis and angle
[47]	Ultrasounds	CORE-TX [48]	Indoor Surveillance Small office with 6 rooms	Abs. Error
[49]	Ultrasounds	Acoustic Beacons	small area 3 × 3	Average error Trajectory
[50]	UWB	IMU; UWB; and Combination	20 × 20 m	Average error Trajectory
[51]	UWB	BeSopon and Decawave EVK1000	12.4 × 9.6 m	Average Error Median Error 90th percentile CDF Trajectory Histograms
[52]	S.C.Sensor	Samsung smartphones and proprietary podometer	3 Buildings	Average Error
[53]	Hybrid Syst.	Sony Xperia Z3 Compact Samsung Galaxy S5	Office space following an open space concept (2600 m^2^ approx)	Average Error Percentile <5 m
[54]	RFID	low cost IMU (Xsens MTi) Samsung Galaxy S2	1 hall (10 × 7.5 m) 1 corridor (50 m)	Average Error Avg. lateral deviation

## Abbreviations

The following abbreviations are used in this manuscript:

AAL	Active and Assisted Living
AP	Access Point
BT	Bluetooth
BLE	Bluetooth Low Energy
CDF	Cumulative Distribution Function
EvAAL	Evaluating Active and Assisted Living
GNSS	Global Navigation Satellite System
IMU	Inertial Measurement Unit
IoT	Internet of Things
IPIN	Indoor Positioning and Indoor Navigation
IPS	Indoor Positioning System
PDF	Probability Density Function
PDR	Pedestrian Dead Reckoning
RFID	Radio Frequency Identification
RMSE	Root Mean Square Error
UWB	Ultra Wide Band
